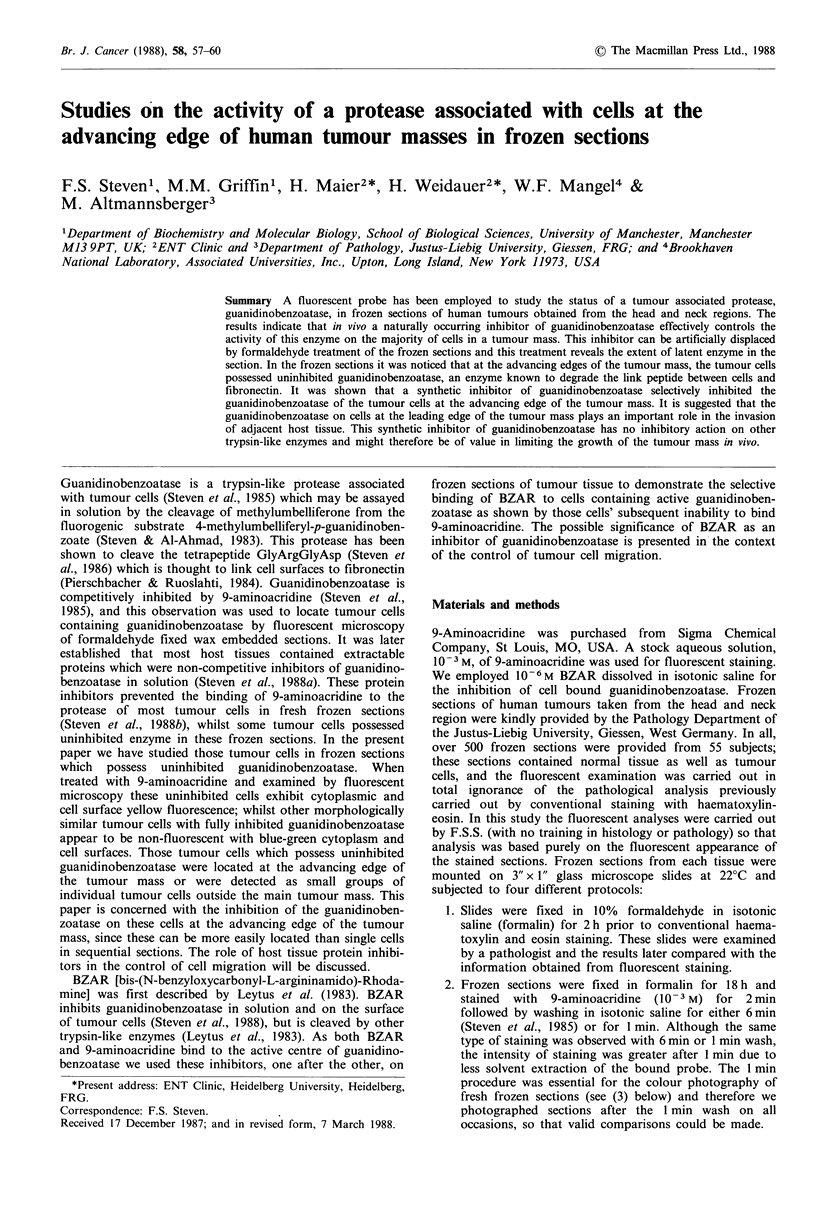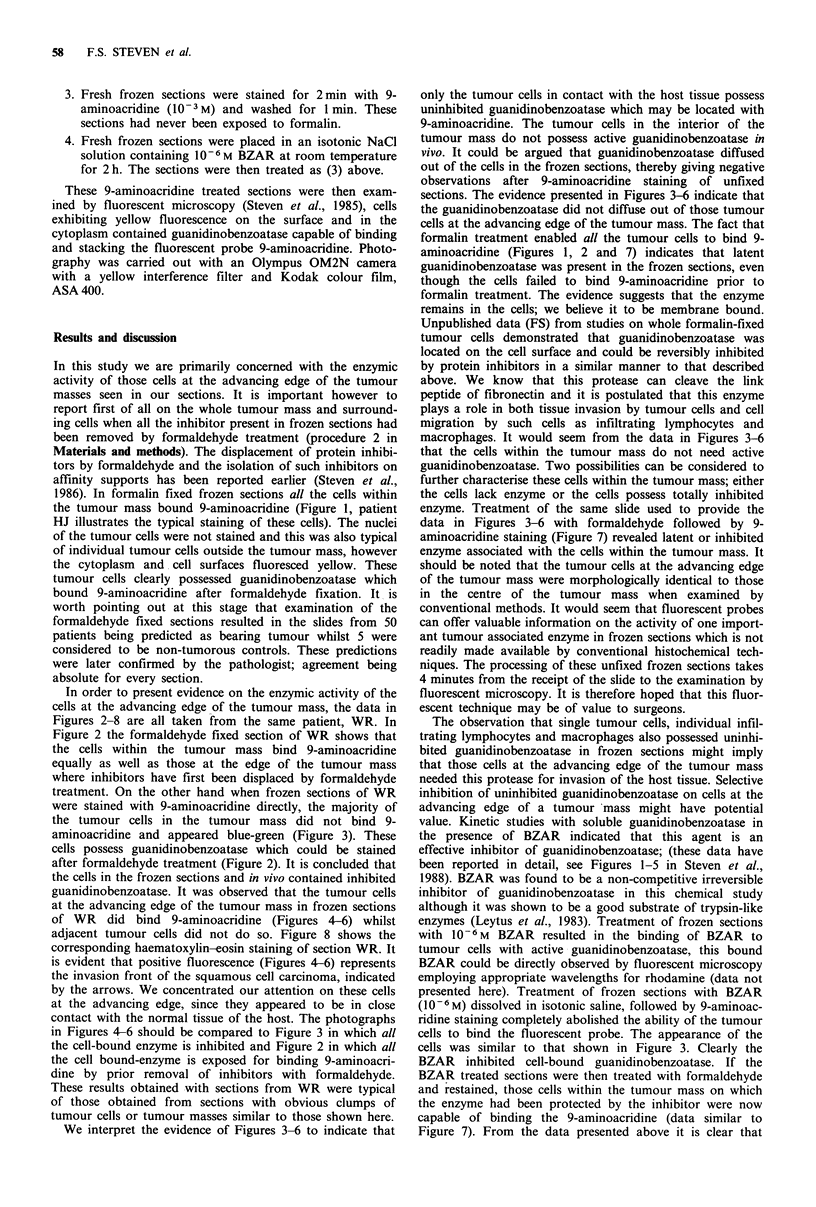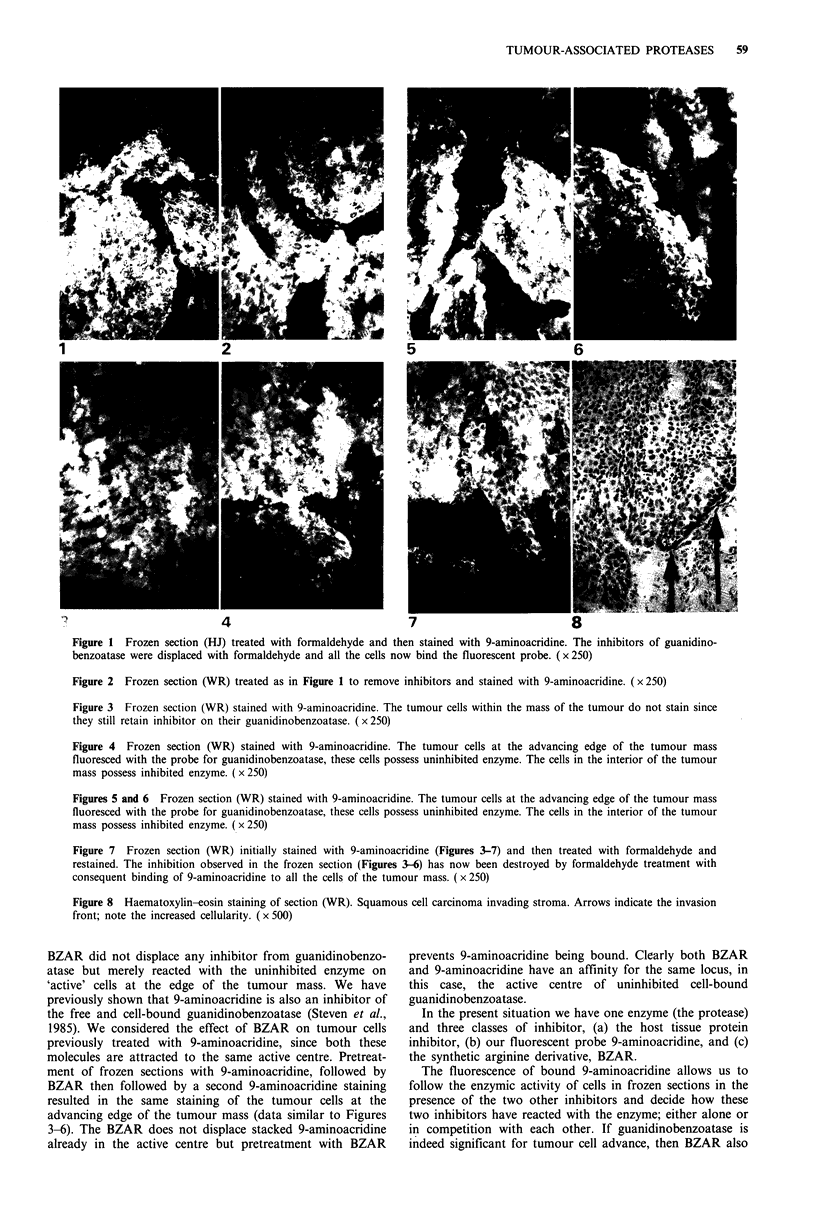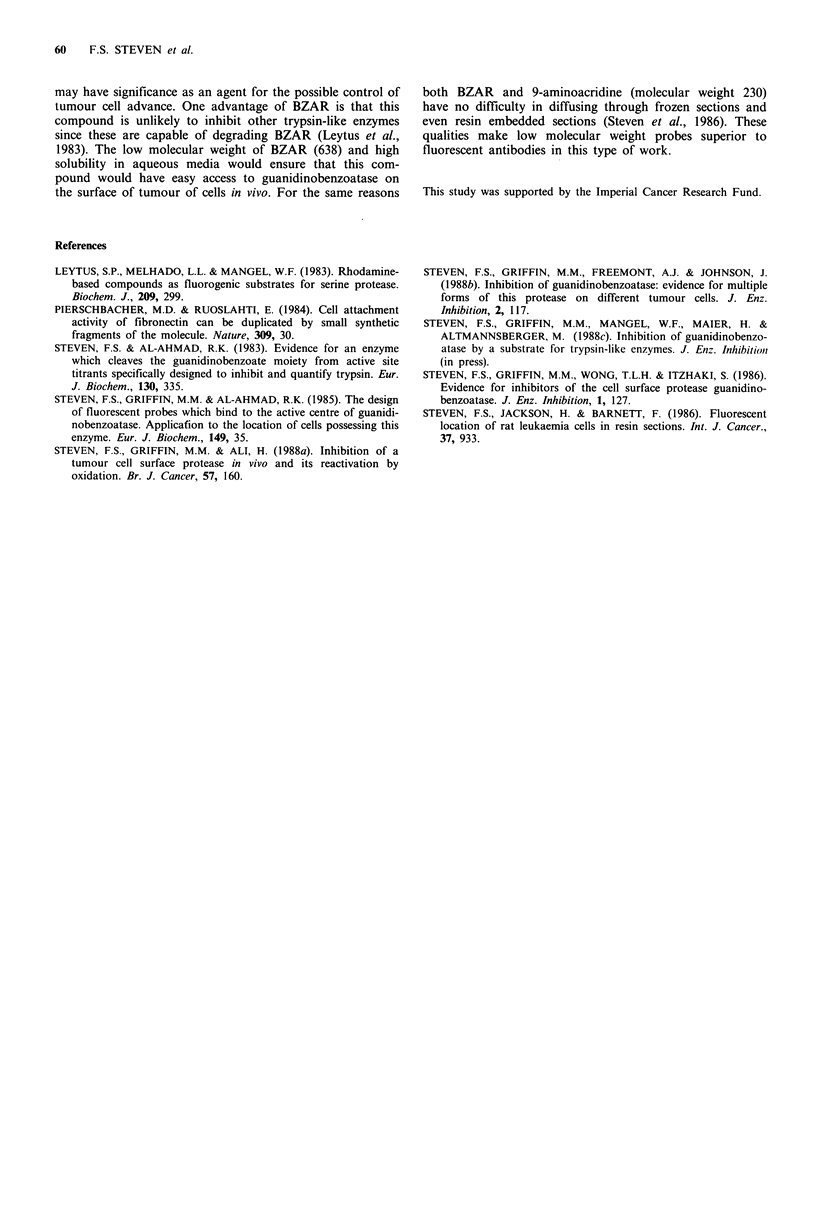# Studies on the activity of a protease associated with cells at the advancing edge of human tumour masses in frozen sections.

**DOI:** 10.1038/bjc.1988.161

**Published:** 1988-07

**Authors:** F. S. Steven, M. M. Griffin, H. Maier, H. Weidauer, W. F. Mangel, M. Altmannsberger

**Affiliations:** Department of Biochemistry and Molecular Biology, School of Biological Sciences, University of Manchester, UK.

## Abstract

**Images:**


*Br. J. Cancer* (1988), **58**, 57–60 © The Macmillan Press Ltd., 1988

### Studies on the activity of a protease associated with cells at the advancing edge of human tumour masses in frozen sections

F.S. Steven[1], M.M. Griffin[1], H. Maier[2]*, H. Weidauer[2]*, W.F. Mangel[4] & M. Altmannsberger[3]

[1]*Department of Biochemistry and Molecular Biology, School of Biological Sciences, University of Manchester, Manchester M13 9PT, UK;* [2]*ENT Clinic and* [3]*Department of Pathology, Justus-Liebig University, Giessen, FRG; and* [4]*Brookhaven National Laboratory, Associated Universities, Inc., Upton, Long Island, New York 11973, USA*

**Summary** A fluorescent probe has been employed to study the status of a tumour associated protease, guanidinobenzoatase, in frozen sections of human tumours obtained from the head and neck regions. The results indicate that *in vivo* a naturally occurring inhibitor of guanidinobenzoatase effectively controls the activity of this enzyme on the majority of cells in a tumour mass. This inhibitor can be artificially displaced by formaldehyde treatment of the frozen sections and this treatment reveals the extent of latent enzyme in the section. In the frozen sections it was noticed that at the advancing edges of the tumour mass, the tumour cells possessed uninhibited guanidinobenzoatase, an enzyme known to degrade the link peptide between cells and fibronectin. It was shown that a synthetic inhibitor of guanidinobenzoatase selectively inhibited the guanidinobenzoatase of the tumour cells at the advancing edge of the tumour mass. It is suggested that the guanidinobenzoatase on cells at the leading edge of the tumour mass plays an important role in the invasion of adjacent host tissue. This synthetic inhibitor of guanidinobenzoatase has no inhibitory action on other trypsin-like enzymes and might therefore be of value in limiting the growth of the tumour mass *in vivo*.

Guanidinobenzoatase is a trypsin-like protease associated with tumour cells (Steven *et al.*, 1985) which may be assayed in solution by the cleavage of methylumbelliferone from the fluorogenic substrate 4-methylumbelliferyl-*p*-guanidinobenzoate (Steven & Al-Ahmad, 1983). This protease has been shown to cleave the tetrapeptide GlyArgGlyAsp (Steven *et al.*, 1986) which is thought to link cell surfaces to fibronectin (Pierschbacher & Ruoslahti, 1984). Guanidinobenzoatase is competitively inhibited by 9-aminoacridine (Steven *et al.*, 1985), and this observation was used to locate tumour cells containing guanidinobenzoatase by fluorescent microscopy of formaldehyde fixed wax embedded sections. It was later established that most host tissues contained extractable proteins which were non-competitive inhibitors of guanidinobenzoatase in solution (Steven *et al.*, 1988*a*). These protein inhibitors prevented the binding of 9-aminoacridine to the protease of most tumour cells in fresh frozen sections (Steven *et al.*, 1988*b*), whilst some tumour cells possessed uninhibited enzyme in these frozen sections. In the present paper we have studied those tumour cells in frozen sections which possess uninhibited guanidinobenzoatase. When treated with 9-aminoacridine and examined by fluorescent microscopy these uninhibited cells exhibit cytoplasmic and cell surface yellow fluorescence; whilst other morphologically similar tumour cells with fully inhibited guanidinobenzoatase appear to be non-fluorescent with blue-green cytoplasm and cell surfaces. Those tumour cells which possess uninhibited guanidinobenzoatase were located at the advancing edge of the tumour mass or were detected as small groups of individual tumour cells outside the main tumour mass. This paper is concerned with the inhibition of the guanidinobenzoatase on these cells at the advancing edge of the tumour mass, since these can be more easily located than single cells in sequential sections. The role of host tissue protein inhibitors in the control of cell migration will be discussed.

BZAR [bis-(N-benzyloxycarbonyl-L-argininamido)-Rhodamine] was first described by Leytus *et al.* (1983). BZAR inhibits guanidinobenzoatase in solution and on the surface of tumour cells (Steven *et al.*, 1988), but is cleaved by other trypsin-like enzymes (Leytus *et al.*, 1983). As both BZAR and 9-aminoacridine bind to the active centre of guanidinobenzoatase we used these inhibitors, one after the other, on frozen sections of tumour tissue to demonstrate the selective binding of BZAR to cells containing active guanidinobenzoatase as shown by those cells' subsequent inability to bind 9-aminoacridine. The possible significance of BZAR as an inhibitor of guanidinobenzoatase is presented in the context of the control of tumour cell migration.

### Materials and methods

9-Aminoacridine was purchased from Sigma Chemical Company, St Louis, MO, USA. A stock aqueous solution, $10^{-3}$ M, of 9-aminoacridine was used for fluorescent staining. We employed $10^{-6}$ M BZAR dissolved in isotonic saline for the inhibition of cell bound guanidinobenzoatase. Frozen sections of human tumours taken from the head and neck region were kindly provided by the Pathology Department of the Justus-Liebig University, Giessen, West Germany. In all, over 500 frozen sections were provided from 55 subjects; these sections contained normal tissue as well as tumour cells, and the fluorescent examination was carried out in total ignorance of the pathological analysis previously carried out by conventional staining with haematoxylin-eosin. In this study the fluorescent analyses were carried out by F.S.S. (with no training in histology or pathology) so that analysis was based purely on the fluorescent appearance of the stained sections. Frozen sections from each tissue were mounted on 3″×1″ glass microscope slides at 22°C and subjected to four different protocols:

1. Slides were fixed in 10% formaldehyde in isotonic saline (formalin) for 2 h prior to conventional haematoxylin and eosin staining. These slides were examined by a pathologist and the results later compared with the information obtained from fluorescent staining.
2. Frozen sections were fixed in formalin for 18 h and stained with 9-aminoacridine ($10^{-3}$ M) for 2 min followed by washing in isotonic saline for either 6 min (Steven *et al.*, 1985) or for 1 min. Although the same type of staining was observed with 6 min or 1 min wash, the intensity of staining was greater after 1 min due to less solvent extraction of the bound probe. The 1 min procedure was essential for the colour photography of fresh frozen sections (see (3) below) and therefore we photographed sections after the 1 min wash on all occasions, so that valid comparisons could be made.

*Present address: ENT Clinic, Heidelberg University, Heidelberg, FRG.

Correspondence: F.S. Steven.

Received 17 December 1987; and in revised form, 7 March 1988.

3. Fresh frozen sections were stained for 2 min with 9-aminoacridine ($10^{-3}$ M) and washed for 1 min. These sections had never been exposed to formalin.
4. Fresh frozen sections were placed in an isotonic NaCl solution containing $10^{-6}$ M BZAR at room temperature for 2 h. The sections were then treated as (3) above.

These 9-aminoacridine treated sections were then examined by fluorescent microscopy (Steven *et al.*, 1985), cells exhibiting yellow fluorescence on the surface and in the cytoplasm contained guanidinobenzoatase capable of binding and stacking the fluorescent probe 9-aminoacridine. Photography was carried out with an Olympus OM2N camera with a yellow interference filter and Kodak colour film, ASA 400.

### Results and discussion

In this study we are primarily concerned with the enzymic activity of those cells at the advancing edge of the tumour masses seen in our sections. It is important however to report first of all on the whole tumour mass and surrounding cells when all the inhibitor present in frozen sections had been removed by formaldehyde treatment (procedure 2 in **Materials and methods**). The displacement of protein inhibitors by formaldehyde and the isolation of such inhibitors on affinity supports has been reported earlier (Steven *et al.*, 1986). In formalin fixed frozen sections *all* the cells within the tumour mass bound 9-aminoacridine (Figure 1, patient HJ illustrates the typical staining of these cells). The nuclei of the tumour cells were not stained and this was also typical of individual tumour cells outside the tumour mass, however the cytoplasm and cell surfaces fluoresced yellow. These tumour cells clearly possessed guanidinobenzoatase which bound 9-aminoacridine after formaldehyde fixation. It is worth pointing out at this stage that examination of the formaldehyde fixed sections resulted in the slides from 50 patients being predicted as bearing tumour whilst 5 were considered to be non-tumorous controls. These predictions were later confirmed by the pathologist; agreement being absolute for every section.

In order to present evidence on the enzymic activity of the cells at the advancing edge of the tumour mass, the data in Figures 2–8 are all taken from the same patient, WR. In Figure 2 the formaldehyde fixed section of WR shows that the cells within the tumour mass bind 9-aminoacridine equally as well as those at the edge of the tumour mass where inhibitors have first been displaced by formaldehyde treatment. On the other hand when frozen sections of WR were stained with 9-aminoacridine directly, the majority of the tumour cells in the tumour mass did not bind 9-aminoacridine and appeared blue-green (Figure 3). These cells possess guanidinobenzoatase which could be stained after formaldehyde treatment (Figure 2). It is concluded that the cells in the frozen sections and *in vivo* contained inhibited guanidinobenzoatase. It was observed that the tumour cells at the advancing edge of the tumour mass in frozen sections of WR did bind 9-aminoacridine (Figures 4–6) whilst adjacent tumour cells did not do so. Figure 8 shows the corresponding haematoxylin–eosin staining of section WR. It is evident that positive fluorescence (Figures 4–6) represents the invasion front of the squamous cell carcinoma, indicated by the arrows. We concentrated our attention on these cells at the advancing edge, since they appeared to be in close contact with the normal tissue of the host. The photographs in Figures 4–6 should be compared to Figure 3 in which *all* the cell-bound enzyme is inhibited and Figure 2 in which *all* the cell bound-enzyme is exposed for binding 9-aminoacridine by prior removal of inhibitors with formaldehyde. These results obtained with sections from WR were typical of those obtained from sections with obvious clumps of tumour cells or tumour masses similar to those shown here.

We interpret the evidence of Figures 3–6 to indicate that only the tumour cells in contact with the host tissue possess uninhibited guanidinobenzoatase which may be located with 9-aminoacridine. The tumour cells in the interior of the tumour mass do not possess active guanidinobenzoatase *in vivo*. It could be argued that guanidinobenzoatase diffused out of the cells in the frozen sections, thereby giving negative observations after 9-aminoacridine staining of unfixed sections. The evidence presented in Figures 3–6 indicate that the guanidinobenzoatase did not diffuse out of those tumour cells at the advancing edge of the tumour mass. The fact that formalin treatment enabled *all* the tumour cells to bind 9-aminoacridine (Figures 1, 2 and 7) indicates that latent guanidinobenzoatase was present in the frozen sections, even though the cells failed to bind 9-aminoacridine prior to formalin treatment. The evidence suggests that the enzyme remains in the cells; we believe it to be membrane bound. Unpublished data (FS) from studies on whole formalin-fixed tumour cells demonstrated that guanidinobenzoatase was located on the cell surface and could be reversibly inhibited by protein inhibitors in a similar manner to that described above. We know that this protease can cleave the link peptide of fibronectin and it is postulated that this enzyme plays a role in both tissue invasion by tumour cells and cell migration by such cells as infiltrating lymphocytes and macrophages. It would seem from the data in Figures 3–6 that the cells within the tumour mass do not need active guanidinobenzoatase. Two possibilities can be considered to further characterise these cells within the tumour mass; either the cells lack enzyme or the cells possess totally inhibited enzyme. Treatment of the same slide used to provide the data in Figures 3–6 with formaldehyde followed by 9-aminoacridine staining (Figure 7) revealed latent or inhibited enzyme associated with the cells within the tumour mass. It should be noted that the tumour cells at the advancing edge of the tumour mass were morphologically identical to those in the centre of the tumour mass when examined by conventional methods. It would seem that fluorescent probes can offer valuable information on the activity of one important tumour associated enzyme in frozen sections which is not readily made available by conventional histochemical techniques. The processing of these unfixed frozen sections takes 4 minutes from the receipt of the slide to the examination by fluorescent microscopy. It is therefore hoped that this fluorescent technique may be of value to surgeons.

The observation that single tumour cells, individual infiltrating lymphocytes and macrophages also possessed uninhibited guanidinobenzoatase in frozen sections might imply that those cells at the advancing edge of the tumour mass needed this protease for invasion of the host tissue. Selective inhibition of uninhibited guanidinobenzoatase on cells at the advancing edge of a tumour mass might have potential value. Kinetic studies with soluble guanidinobenzoatase in the presence of BZAR indicated that this agent is an effective inhibitor of guanidinobenzoatase; (these data have been reported in detail, see Figures 1–5 in Steven *et al.*, 1988). BZAR was found to be a non-competitive irreversible inhibitor of guanidinobenzoatase in this chemical study although it was shown to be a good substrate of trypsin-like enzymes (Leytus *et al.*, 1983). Treatment of frozen sections with $10^{-6}$ M BZAR resulted in the binding of BZAR to tumour cells with active guanidinobenzoatase, this bound BZAR could be directly observed by fluorescent microscopy employing appropriate wavelengths for rhodamine (data not presented here). Treatment of frozen sections with BZAR ($10^{-6}$ M) dissolved in isotonic saline, followed by 9-aminoacridine staining completely abolished the ability of the tumour cells to bind the fluorescent probe. The appearance of the cells was similar to that shown in Figure 3. Clearly the BZAR inhibited cell-bound guanidinobenzoatase. If the BZAR treated sections were then treated with formaldehyde and restained, those cells within the tumour mass on which the enzyme had been protected by the inhibitor were now capable of binding the 9-aminoacridine (data similar to Figure 7). From the data presented above it is clear that

1

2

5

6

3

4

7

8

**Figure 1** Frozen section (HJ) treated with formaldehyde and then stained with 9-aminoacridine. The inhibitors of guanidinobenzoatase were displaced with formaldehyde and all the cells now bind the fluorescent probe. (×250)

**Figure 2** Frozen section (WR) treated as in **Figure 1** to remove inhibitors and stained with 9-aminoacridine. (×250)

**Figure 3** Frozen section (WR) stained with 9-aminoacridine. The tumour cells within the mass of the tumour do not stain since they still retain inhibitor on their guanidinobenzoatase. (×250)

**Figure 4** Frozen section (WR) stained with 9-aminoacridine. The tumour cells at the advancing edge of the tumour mass fluoresced with the probe for guanidinobenzoatase, these cells possess uninhibited enzyme. The cells in the interior of the tumour mass possess inhibited enzyme. (×250)

**Figures 5 and 6** Frozen section (WR) stained with 9-aminoacridine. The tumour cells at the advancing edge of the tumour mass fluoresced with the probe for guanidinobenzoatase, these cells possess uninhibited enzyme. The cells in the interior of the tumour mass possess inhibited enzyme. (×250)

**Figure 7** Frozen section (WR) initially stained with 9-aminoacridine (**Figures 3–7**) and then treated with formaldehyde and restained. The inhibition observed in the frozen section (**Figures 3–6**) has now been destroyed by formaldehyde treatment with consequent binding of 9-aminoacridine to all the cells of the tumour mass. (×250)

**Figure 8** Haematoxylin–eosin staining of section (WR). Squamous cell carcinoma invading stroma. Arrows indicate the invasion front; note the increased cellularity. (×500)

BZAR did not displace any inhibitor from guanidinobenzoatase but merely reacted with the uninhibited enzyme on 'active' cells at the edge of the tumour mass. We have previously shown that 9-aminoacridine is also an inhibitor of the free and cell-bound guanidinobenzoatase (Steven *et al.*, 1985). We considered the effect of BZAR on tumour cells previously treated with 9-aminoacridine, since both these molecules are attracted to the same active centre. Pretreatment of frozen sections with 9-aminoacridine, followed by BZAR then followed by a second 9-aminoacridine staining resulted in the same staining of the tumour cells at the advancing edge of the tumour mass (data similar to Figures 3–6). The BZAR does not displace stacked 9-aminoacridine already in the active centre but pretreatment with BZAR prevents 9-aminoacridine being bound. Clearly both BZAR and 9-aminoacridine have an affinity for the same locus, in this case, the active centre of uninhibited cell-bound guanidinobenzoatase.

In the present situation we have one enzyme (the protease) and three classes of inhibitor, (a) the host tissue protein inhibitor, (b) our fluorescent probe 9-aminoacridine, and (c) the synthetic arginine derivative, BZAR.

The fluorescence of bound 9-aminoacridine allows us to follow the enzymic activity of cells in frozen sections in the presence of the two other inhibitors and decide how these two inhibitors have reacted with the enzyme; either alone or in competition with each other. If guanidinobenzoatase is indeed significant for tumour cell advance, then BZAR also may have significance as an agent for the possible control of tumour cell advance. One advantage of BZAR is that this compound is unlikely to inhibit other trypsin-like enzymes since these are capable of degrading BZAR (Leytus *et al.*, 1983). The low molecular weight of BZAR (638) and high solubility in aqueous media would ensure that this compound would have easy access to guanidinobenzoatase on the surface of tumour of cells *in vivo*. For the same reasons both BZAR and 9-aminoacridine (molecular weight 230) have no difficulty in diffusing through frozen sections and even resin embedded sections (Steven *et al.*, 1986). These qualities make low molecular weight probes superior to fluorescent antibodies in this type of work.

This study was supported by the Imperial Cancer Research Fund.

### References

LEYTUS, S.P., MELHADO, L.L. & MANGEL, W.F. (1983). Rhodamine-based compounds as fluorogenic substrates for serine protease. *Biochem. J.*, **209**, 299.

PIERSCHBACHER, M.D. & RUOSLAHTI, E. (1984). Cell attachment activity of fibronectin can be duplicated by small synthetic fragments of the molecule. *Nature*, **309**, 30.

STEVEN, F.S. & AL-AHMAD, R.K. (1983). Evidence for an enzyme which cleaves the guanidinobenzoate moiety from active site titrants specifically designed to inhibit and quantify trypsin. *Eur. J. Biochem.*, **130**, 335.

STEVEN, F.S., GRIFFIN, M.M. & AL-AHMAD, R.K. (1985). The design of fluorescent probes which bind to the active centre of guanidinobenzoatase. Applicafion to the location of cells possessing this enzyme. *Eur. J. Biochem.*, **149**, 35.

STEVEN, F.S., GRIFFIN, M.M. & ALI, H. (1988*a*). Inhibition of a tumour cell surface protease *in vivo* and its reactivation by oxidation. *Br. J. Cancer*, **57**, 160.

STEVEN, F.S., GRIFFIN, M.M., FREEMONT, A.J. & JOHNSON, J. (1988*b*). Inhibition of guanidinobenzoatase: evidence for multiple forms of this protease on different tumour cells. *J. Enz. Inhibition*, **2**, 117.

STEVEN, F.S., GRIFFIN, M.M., MANGEL, W.F., MAIER, H. & ALTMANNSBERGER, M. (1988*c*). Inhibition of guanidinobenzoatase by a substrate for trypsin-like enzymes. *J. Enz. Inhibition* (in press).

STEVEN, F.S., GRIFFIN, M.M., WONG, T.L.H. & ITZHAKI, S. (1986). Evidence for inhibitors of the cell surface protease guanidinobenzoatase. *J. Enz. Inhibition*, **1**, 127.

STEVEN, F.S., JACKSON, H. & BARNETT, F. (1986). Fluorescent location of rat leukaemia cells in resin sections. *Int. J. Cancer.*, **37**, 933.